# Single hair analysis by X-ray fluorescence spectrometry detects small changes in dietary zinc intake: A nested randomized controlled trial

**DOI:** 10.3389/fnut.2023.1139017

**Published:** 2023-03-24

**Authors:** Christopher J. Frederickson, David E. B. Fleming, Dan Asael, Mukhtiar Zaman, Randal Ferguson, Michaela G. Kaiser, Bryn D. Rankin, Kassia M. M. Schenkels, Andrew I. T. Hess, Andrianna R. Scott, Cathleen J. M. Frederickson, Jonathan K. Sinclair, Nicola M. Lowe

**Affiliations:** ^1^NeuroBioTex, Inc., Galveston, TX, United States; ^2^Physics Department, Mount Allison University, Sackville, NB, Canada; ^3^Department of Earth and Planetary Sciences, Yale University, New Haven, CT, United States; ^4^Pulmonology Department, Rehman Medical College, Peshawar, Khyber Pakhtunkhwa, Pakistan; ^5^UCLan Research Centre for Global Development, University of Central Lancashire, Preston, Lancashire, United Kingdom

**Keywords:** zinc, hair, X-ray fluorescence spectrometry, zinc biomarker, zinc biofortification

## Abstract

The aim of this study was to determine whether X-ray fluorescence spectrometry (XRF) could be used to detect changes in hair zinc concentration in response to a modest daily increase in zinc from the consumption of zinc biofortified wheat flour. This study was conducted as part of an effectiveness trial (BiZiFED2) exploring the potential for zinc biofortified wheat to alleviate zinc deficiency in adolescent girls aged 10–16 years in Pakistan (trial registration ID ISRCTN17107812). A randomized controlled design was used. Participants received either control flour or zinc biofortified flour for 6 months. Consumption of biofortified flour resulted in an average daily increase in dietary zinc intake of 1.5 mg per day above that of the control flour. At baseline and at the end of the intervention, individual hair samples (control: *n* = 59, intervention: *n* = 64) were analyzed for zinc and sulfur content by XRF. Data were analyzed using linear mixed effects models to contrast between trial groups the changes from baseline to end point and also to compare baseline and end point values within each trial group. Increases from baseline to endpoint in both sulfur and zinc were significantly greater in the intervention group compared to control (sulfur counts. Control: baseline = 119.87 ± 20.33 and endpoint = 121.58 ± 23.58/intervention: baseline = 122.67 ± 24.19 and endpoint = 131.60 ± 21.34); (Zinc counts. Control: baseline = 50.88 ± 14.33 and endpoint = 54.82 ± 14.61/intervention: baseline = 49.61 ± 10.77 and endpoint = 58.79 ± 12.20). For these parameters, there were also significant increases from baseline to endpoint in the intervention group but not in control. Furthermore, for Zn:S count ratio there were no differences in terms of the magnitude of the change from baseline to endpoint in the control group, although significant increases from baseline to endpoint were evident in the intervention group (Zn:S count ratio. Control: baseline = 0.42 ± 0.10 and endpoint = 0.45 ± 0.08/intervention: baseline = 0.41 ± 0.08 and endpoint = 0.45 ± 0.08). A modest increase in dietary zinc over 6 months resulted in a detectable increase in both sulfur and zinc counts in individual hairs measured using XRF. This offers a sensitive, non-invasive method to monitor changes within subjects in response to dietary zinc interventions.

## 1. Introduction

It has been clear since the pioneering work of Prasad and Sandstead (1, 2) that zinc deficiency is a global public health problem, which if left untreated may result in stunted growth and impaired neurodevelopment in children, increased susceptibility to infections in children and adults, and complications during pregnancy and childbirth (3). It is estimated that over 17% of the global population are at risk of inadequate zinc intake with the greatest burden of deficiency in low- and middle-income countries (LMICs) (4). Zinc is a type 2 nutrient, meaning that a diverse range of biochemical functions are affected by zinc deficiency, with non-specific physiological symptoms. In contrast, for type 1 nutrients such as iron, deficiency is linked to specific measurable functions (5). This poses a challenge for diagnosis of the zinc status of an individual, and the monitoring of responses to dietary zinc interventions such as fortification, supplementation, and biofortification.

Numerous potential biomarkers of zinc status in humans have been explored and evaluated in zinc supplementation and depletion trials. Systematic reviews and meta-analyses of the data have concluded that in healthy individuals, the concentration of zinc plasma, serum, urine, nails, and hair respond reliably to changes in dietary zinc intake (6–8). However, for many putative biomarkers, there were insufficient data at the time of the review to fully evaluate their potential. Serum or plasma zinc concentration has for a long time been the most widely used zinc biomarker in human studies, however its limitations when used outside of a carefully controlled trial are well-documented (9). For example, plasma zinc concentration falls if inflammation is present as part of the acute phase response, potentially leading to a misdiagnosis of zinc deficiency or over estimation of the prevalence of zinc deficiency in a population study where the burden of infection is high. This can be adjusted for by measuring the acute phase proteins, C-reactive protein (CRP) and α-1-acid glycoprotein (AGP), alongside plasma zinc concentration and either excluding observations where infection is concurrent for population studies or applying a correction factor to individual values (10). However, the sensitivity of plasma zinc concentration to small changes in dietary zinc intake, such as those achieved in zinc biofortification trials, is poor. A meta-analysis of pooled data from zinc intervention studies revealed that for every doubling in zinc intake in adults, the difference in serum or plasma zinc concentration was only 6% (11).

Meta-analysis of data from zinc supplementation trials revealed that concentration of zinc in a 24-h urine sample increased in response to increases in dietary zinc intake. The practicalities involved in collecting a full 24-h sample outside of a controlled setting are daunting, and a simpler approach is to collect a random “spot” urine sample, and adjust for hydration status, using creatinine, osmolarity, or specific gravity. However, even after these adjustments, urinary zinc concentration does not correlate well with plasma zinc concentration from the same individual in large population studies, casting doubt on its usefulness for individual assessment in free-living situations (12).

Fingernail (or toenail) clippings have been studied extensively as biomarkers for nutrient minerals as well as toxic metals (13–16). Many careful studies have shown a strong and highly significant relationship between an individuals' past zinc intake and their nail zinc concentration in the portion of the nail that was growing during the period of intake that was observed (17). However, the time lag between the growth of a nail segment (in the growth cone, under the cuticle) and the availability for clipping varies from about 5–18 months depending on the length of the toenail or fingernail (18). Thus, nail clippings reflect a zinc status that is retrospective and does not show recent changes in the individual's zinc intake.

Hair zinc concentration has also been measured in human supplementation and depletion trials and has been shown to respond to changes in dietary zinc intake (19, 20) although this finding is inconsistent and controversial (13, 21). The analysis, which has the advantage of being relatively non-invasive, involves taking a hair sample (20+ strands) cut very close to the scalp and not more that 5–10 mm in length (most recent growth). Conventional assays then require acid digestion and zinc content determination by AAS or mass spectrometry. Potential contamination from hair products (shampoos, dyes, gels, etc.) must be avoided, and surface contamination removed by washing the sample in distilled water prior to analysis (20). The sample preparation and analysis requires specialized laboratory setting, and is time consuming.

To simplify the analysis of zinc in easily accessible human tissue samples, the method of X-ray fluorescence (XRF) spectrometry has been developed recently as a fast and minimally invasive technique. Results from XRF spectrometry have correlated well with “gold standard” analytical techniques when measuring zinc and arsenic (15, 22) in human toenail clippings. For decades, the XRF method has been used to measure lead in human bones *in vivo* (23). In the present study, we have adapted the XRF technique for the measurement of zinc in single strands of human hair.

The aim of this study was to determine whether a modest average daily increase in zinc intake of 1.5 mg per day for 6 months, from the consumption of zinc biofortified flour, could be detected using XRF in the hair of adolescent girls participating in a randomized controlled trial (RCT) (24).

## 2. Materials and methods

### 2.1. Study setting and design

This study was conducted as part of a large, double blind, cluster-randomized controlled trial, designed to examine the effectiveness of consuming zinc biofortified wheat flour in adolescent girls and children aged <5 years, living a rural location in northwest Pakistan (24). The study was conducted between November 2019 and March 2021. Ethical approval was granted from the University of Central Lancashire STEMH Ethics Committee (reference number: STEMH 1014) and Khyber Medical University Ethics Committee (reference number: DIR/KMU-EB/BZ/000683). The study was registered with the ISRCTN registry (Trial registration number ISRCTN17107812). Although the larger RCT (*n* = 517 adolescent girls) was cluster randomized, the subsample of participants represented in this report (*n* = 123) were randomly selected, based on the quality of the hair sample by study arm, irrespective of the cluster. Therefore, reporting of this study complies with the Consolidated Standards of Reporting Trials (CONSORT) guidelines for standard randomized analyses rather than cluster randomized analyses (25, 26).

The complete protocol, including the study setting, subject recruitment and consent process, data monitoring process to review any adverse events and intervention randomization procedures, have previously been described in detail (24) with some deviations to original protocol due to the COVID19 pandemic described subsequently (27). Households were recruited to participate in the study. Eligibility criteria were defined as households with at least one unmarried, non-pregnant, non-lactating adolescent girl (10–16 years), and one child (1–5 years). The target was to recruit 500 adolescent-child pairs, powered for the primary outcome measure for the trial which was plasma zinc concentration. This was achieved after successfully recruiting 483 eligible households, arranged in 28 clusters. All households received standard wheat flour in a 10.5-month equilibration phase (extended from 6 months due to safety concerns regarding exposure to COVID19 during the pandemic) at the end of which (August–September 2020) baseline blood and hair samples were collected from the adolescent girls only. This was followed by the intervention period in which households were provided with either standard control flour (SCF) or zinc biofortified flour (ZBF) for 25 weeks according to the cluster randomization protocol (24). The zinc content of the ZBF and SCF was 20.7± 5.6 and 17.0 ± 2.6 mg/kg, respectively. Based on a typical daily flour consumption of 405 g/day, the ZBF provided an estimated additional daily zinc intake of 1.5 mg (27). Blood and hair samples were collected again from the adolescent girls at the end of the intervention phase (March 2021).

### 2.2. Sample collection and analysis

Hair samples were collected from the adolescent girls at the same time as the blood samples. Girls were invited to remove their head covering in private, behind a screen. They were provided with a stainless-steel comb and a new, self-sealing plastic bag and asked to comb their hair, and place any hairs that came away by the root into the plastic bag. The bags were labeled with participant ID and date of collection and stored in a cool dry place before shipment to the USA and Canada for analysis. A total of 123 pairs (baseline and endpoint) of hair samples (control: *n* = 59 pairs, intervention: *n* = 64 pairs) were analyzed for zinc and sulfur content by XRF. Human hair is comprised of keratin which is rich in the sulfur containing amino acid, cysteine, therefore sulfur was considered a valid proxy for total protein content. Samples were labeled with unique ID numbers such that the analysts were blind which arm of the study the participant had been allocated to.

The primary XRF sample analysis was undertaken at NeuroBiotex, Texas, USA (Dr. Chris Frederickson). The first step in analysis of hairs was to examine the collected hair from an individual participant and identify intact strands that had a reasonably complete follicle or “root” on the proximal end. The follicles were then removed with a scalpel, leaving the hair shafts intact but with no follicle. One of the selected hair strands was then mounted between two clear polypropylene sheets on an XRF “cassette” and the cassette was then placed in the XRF, with the proximal end of the hair shaft (1–2 mm distal to the “root”) positioned over the ~1 mm diameter XRF excitation beam path. Sequential measurements were then undertaken with the instrument (XOS, East Greenbush, NY, USA), operated in benchtop mode and set to standard measurement parameters, with “wood” as the chosen matrix, and all three excitation beams employed for 1 min each. No “ppm” result was available with the small mass of one hair strand, thus we recorded and analyzed the total fluorescent “counts” for zinc and for sulfur at each measurement point and recorded the Kalpha (Kα) peak emission for both zinc and sulfur. These counts were taken directly from the analyzer output, corresponding to characteristic X-ray energies (Kα) of zinc and sulfur, respectively. The hair strand was advanced by hand 1 mm in the distal direction after each reading. In this fashion, a total of five measurement points were analyzed for each strand of hair. The mean peak zinc and sulfur counts obtained for these five readings were calculated.

### 2.3. Statistical analysis

Total zinc and sulfur counts determined by XRF, and the Zn:S count ratio from hair samples taken from the participants were expressed as means and standard error of the mean. Outliers were identified as values that were greater than 1.5x interquartile range. The final dataset was comprised of 59 paired hair samples from the control arm and 64 paired hair samples from the intervention arm. Furthermore, to contrast the magnitude of the changes in the aforementioned variables from baseline-endpoint between the two trial arms, values at endpoint were examined using linear mixed-effects models, with trial arm included as a fixed factor and participants included as a random effect, whilst adjusting the model for values at baseline. We undertook these analyses on an intention-to-treat basis and adopted the restricted maximum-likelihood method. In addition, to determine whether main effects of time were present in each trial arm, repeated measures linear mixed models were used to compare baseline and end point values in each trial arm with time (i.e., baseline–endpoint) modeled as a fixed factor and participants included as a random effect. Finally, changes from baseline-endpoint for each experimental parameter were used to create binary variables, i.e., improve/did not improve for each participant. Pearson chi-square tests of independence were used to undertake bivariate cross-tabulation comparisons between the two trial arms, specifically to test differences in the number of participants who exhibited improvements in the experimental outcomes. Probability values for all chi-square analyses in this trial were calculated using Monte-Carlo simulation. For linear mixed models, linear regression coefficients (*β*) and 95% confidence intervals (CI) are reported. Data were analyzed using SPSS v27 (IBM, USA), and the *P*-value for statistical significance was set a priori at the 0.05 level.

## 3. Results

**Figure 1:**
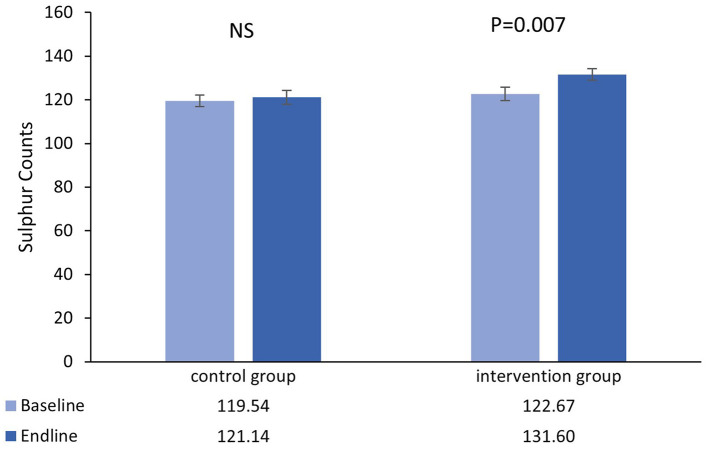
Mean (±SEM) sulfur counts in single hairs collected at the baseline and endline of the control and intervention period.

**Figure 2:**
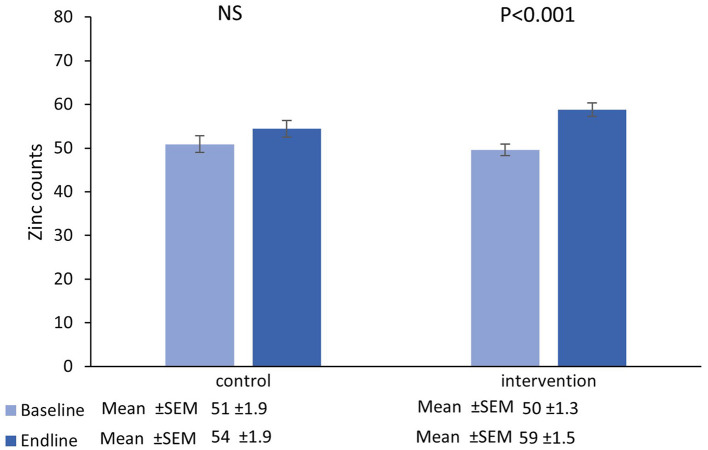
Mean (±SEM) zinc counts in single hairs collected at the baseline and endline of the control and intervention period.

**Figure 3:**
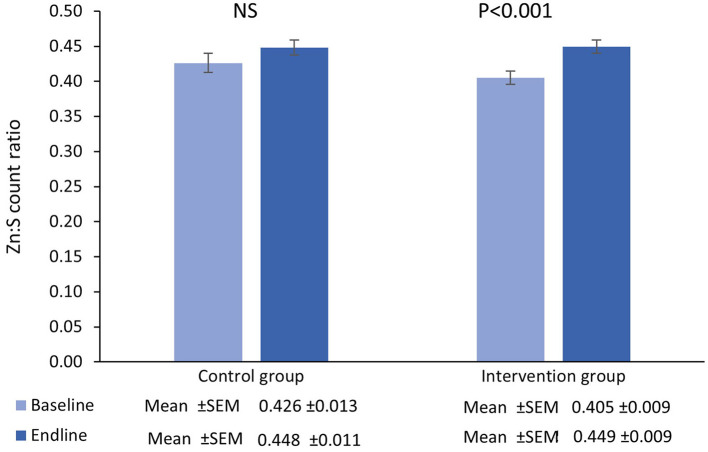
Mean (±SEM) Zn:S count ratio in single hairs collected at the baseline and endline of the control and intervention period.

The number of participants that completed the main trial, together with reasons for drop-out has been previously reported along with the primary outcome measure and no adverse events associated with the intervention were reported (27). The results presented here relate only to the analysis of hair samples randomly selected from the study cohort as described above. The mean and standard error of the mean zinc and sulfur counts recorded at the spectral peak for each hair sample, according to the study arm (outliers removed), are presented in Figures 1, 2, with the corresponding mean Zn:S ratios in Figure 3.

### 3.1. Sulfur

There was no main effect of time in the control group [*β* = 1.61 _(95%CI=−4.97−8.18)_, *t* = 0.49, *P* = 0.627], however there was a significant increase from baseline-endpoint in the intervention group [*β* = 8.93 _(95%CI=2.51−15.35)_, *t* = 2.78, *P* = 0.007]. Furthermore, adjusted for baseline values, increases in the intervention group were significantly greater than those in the control group [*β* = 9.31 _(95%CI=1.72−16.91)_, *t* = 2.43, *P* = 0.017].

### 3.2. Zinc

There was no main effect of time in the control group [*β* = 3.93 _(95%CI=−0.28−8.15)_, *t* = 1.87, *P* = 0.07], however there was a significant increase from baseline-endpoint in the intervention group [*β* = 9.18 _(95%CI=6.06−12.30)_, *t* = 5.88, *P* < 0.001]. Furthermore, adjusted for baseline values, increases in the intervention group were significantly greater than those in the control group [*β* = 4.50 _(95%CI=0.07−8.94)_, *t* = 2.01, *P* = 0.047].

### 3.3. Zn:S ratio

There was no main effect of time in the control group [*β* = 0.02 _(95%CI=−0.003−0.05)_, *t* = 1.71, *P* = 0.09] however there was a significant increase from baseline-endpoint in the intervention group [*β* = 0.04 _(95%CI=0.03−0.06)_, *t* = 4.71, *P* < 0.001]. Furthermore, adjusted for baseline values, there were no significant differences between groups in terms of the magnitude of the change from baseline to endpoint [*β* = 0.009 _(95%CI=−0.02−0.03)_, *t* = 0.71, *P* = 0.48].

Chi-squared analyses showed that the proportion of participants exhibiting increases in sulfur counts did not differ between trial arms [$X_{\left(1\right)}^{2}$ = 0.30, *P* = 0.581]. However, for Zinc [$X_{\left(1\right)}^{2}$ = 7.00, *P* = 0.008] and Zn:S ratio [$X_{\left(1\right)}^{2}$ = 5.88, *P* = 0.015] the proportion of participants exhibiting increases was significantly greater in the intervention group (Table 1).

**Table 1:** Number (%) of participants in each group that had elevated hair Zn counts, S counts, and Zn:S count ratios at the endpoint compared with baseline.

	**Control group (%)**	**Intervention group (%)**
Zinc counts	57.6	79.7
Sulfur counts	57.6	62.5
Zn:S ratio	55.9	76.6

## 4. Discussion

This study has demonstrated that a modest increase in zinc intake of 1.5 mg per day over 25 weeks resulted in a detectable increase in the Zn:S ratio in individual hairs, measured using XRF spectrometry. To our knowledge, this is the first time that XRF analysis of a single hair strand has been used to explore the response to small increases in zinc intake, conducted in a free-living, community setting. It may be noted that this small increase in dietary zinc intake was not reflected in a significant change in plasma zinc concentration measured as part of the broader BiZiFED2 RCT (27).

The structure of hair has been well-characterized. The adult human hair is approximately 20–180 μm in width and consists of a central medulla region, surrounded by the cortex, which is main, fibrous part of the hair, rich in filament forming family of proteins referred to as keratin. The cortex is surrounded by the protective cuticle a layer, comprised of overlapping dead cells and the whole structure is bound together by a lipoprotein membrane (28). Zinc is the most abundant mineral in hair.

Huang et al. compared normal values (mean ± SD) for the zinc concentration in hair from studies conducted in adults and children around the world (29). Values ranged from 129 to 156 μg/g. The considerable variation is due to physiological variables such as color, age, gender, and ethnicity, as well as environmental factors including diet, pollution, and season of the year, all of which may influence growth rate and incorporation of minerals into the hair. The suggested cut off value for hair zinc concentration that is used as an indicator of sub-optimal zinc status in children is <70 μg/g in the spring and summer, or <110 μg/g in the winter (20).

There is evidence from murine and human studies that zinc supplements modulate the width of hair shafts and the speed and cycle of growth (30, 31) so, changes in zinc concentration can be confounded with changes in thickness of the hair strand, its' mass, and also by the timing of hair growth. Thus, mineral contents may be reduced in concentration while being increased in total mass in the same section of emerging hair.

In the present study, sulfur was used as a proxy for keratin, and thus the Zn:S ratio provided adjustment for the protein content and mass of the hair. The data showed a significant increase in the sulfur counts in the hair from intervention group at the endpoint compared to baseline, suggesting that the protein content, and thus the mass of the hair, was also greater at the end of the 25-week intervention period. Despite this, the Zn:S ratio was also significantly greater within the intervention group at the endpoint compared with baseline, indicating an increase in uptake of zinc into the hair filaments above that which is accounted for by the increase in mass. The thickness of the hair strand would be expected to influence the number of counts detected from zinc. All else being equal, a thicker hair would produce more zinc counts. The normalization of zinc counts with respect to sulfur counts largely removes this thickness dependence, and the Zn:S ratio is therefore a more robust indicator of zinc concentration in the hair.

In the control group, although the overall mean zinc, sulfur, and Zn:S ratio did not change significantly, looking at the individual data, just over half of the hair samples had higher Zn and S counts at the end of the study (Table 1). This may be due to seasonal effects as the baseline samples were collected at the end of the summer and endline samples collected in early spring.

## 5. Strengths and limitations

A strength of this study is that it was nested within a randomized cluster-controlled trial. The sample population was relatively homogenous, comprised of non-pregnant, non-lactating girls aged 10–16 of the same ethnicity. The overall prevalence of zinc deficiency among the study participants was high at 68.8%, based on a plasma zinc concentration cut off of <650 μg/L (27). This reflects the predominantly vegetarian diet consumed by the community, which is low in zinc and has a high phytate content which adversely affects zinc bioavailability. Thus, the modest 1.5 mg/d average increase in dietary zinc provided by the zinc biofortified flour constituted a moderate increase in total daily zinc intake of 21%.

Another strength of this study is that, to the best of our knowledge, this is the first study to explore XRF as a means of determining the zinc content of a single hair strand. A limitation to this method is that the values obtained are not fully quantitative, meaning that they require calibration against a quantitative technique in order to convert the zinc counts to zinc content per gram of hair. In addition, the mode of data collection by XRF requires further work to optimize the instrument algorithms, data capture and reproducibility between laboratories. The XRF instrument at Neurobiotex (Dr. Chris Frederickson) was configured to record the value spectral peak for zinc and sulfur. An alternative approach is to integrate the area under all available peaks to provide a value for the total counts for each element. We explored the correlation between these two modes of data collection by analyzing duplicate hair strands (*n* = 36) by XRF at Mount Allison University, Canada (MTA, Dr. David Fleming). The XRF system used was the same as that used at Neurobiotex (HD Mobile unit (XOS, East Greenbush, NY, USA), operated in benchtop mode. The total number of counts for zinc and for sulfur was determined at each of five measurement points along the hair, 1 mm apart. For the first 16 samples the MTA instrument was set to count for 5 min in total. For the second set of 20, this was decreased to 3 min to more closely match the protocol used at Neurobiotex. The correlations for the zinc and sulfur are provided in Supplementary Figures S1, S2. Zinc and sulfur counts correlated well for 5-min collection time (sulfur: *R*^2^ = 0.853, zinc *R*^2^ = 0.853) but was reduced when the collection time was adjusted to 3 min (sulfur: *R*^2^ = 0.264, zinc *R*^2^ = 0.468). It is unlikely the differences in correlation were strongly related to the change in collection time, as shown in Supplementary Figure S3, which shows a typical 5 min spectrum and typical 3 min spectrum for comparison. This demonstrates that the 3 min measurement is not noticeably more noisy and has clear detection peaks. A more likely explanation is that the consistency of positioning the hairs within the X-ray beam may have varied slightly between the two different labs over time. This explanation would suggest Zn:S ratio as a more robust indicator of true zinc concentration. Use of an internal standard in future studies would help to resolve this issue.

A limitation of the study is the XRF alone does not provide a quantitative measure of hair zinc concentration in μg/g hair. To achieve this, calibration of XRF is required, using empirical methods, through comparison with a gold standard technique such as stable isotope dilution mass spectrometry (SIDMS). To that end, we undertook a preliminary investigation of the relationship between XRF generated data and hair zinc concentration (μg/g) measured using SIDMS (Dr. Dan Asael, Yale University). These measurements were undertaken in the 16 hair samples, analyzed at both Neurobiotex and MTA. We explored the correlation between the XRF generated Zn:S count ratios, measured using both the peak and integrative methods, against zinc concentration in the same hair strands measured by SIDMS. The correlations are provided in Supplementary Figures S4, S5. There were significant technical challenges in obtaining accurate weights for the individual hair strands, leading to uncertainty in the SIDMS-determined zinc concentrations of individual hairs. However, despite this, and the small sample size (*n* = 14 hair strands after two outliers were removed) the correlation of SIDMS and Zn:S count ratio using the peak method (*R*^2^ = 0.407) and integrative method (*R*^2^ = 0.304) provided reasonable confidence that calibration at these low levels of zinc detection from a single hair could be achieved in future work.

## 6. Conclusion

This study has demonstrated that single hair analysis by XRF offers a sensitive, non-invasive method to monitor changes within subjects in response to dietary Zn. This has potential for evaluation of the impact of dietary zinc interventions to alleviate zinc deficiency, particularly since the XRF system used in this study is portable and could be used for hair zinc analysis in the field. Hair zinc analysis has the additional advantage over blood-based biomarkers that hair is easily stored and transported. Further work is needed to establish a calibration curve for the conversion of Zn:S ratio data to μg zinc per g hair.

## Data availability statement

The raw data supporting the conclusions of this article will be made available by the authors, without undue reservation.

## Ethics statement

The studies involving human participants were reviewed and approved by Ethical approval was granted from the University of Central Lancashire STEMH Ethics Committee (reference number: STEMH 1014) and Khyber Medical University Ethics Committee (reference number: DIR/KMU-EB/BZ/000683). Written informed consent to participate in this study was provided by the participants' legal guardian/next of kin.

## Author contributions

NML, CJF, and DEBF drafted the manuscript. NML was PI the BiZiFED trial. MZ had oversight of the implementation of the study in Pakistan. Statistical analyses were performed by JKS. XRF analyses were undertaken at Neurobiotex by RF and CJF and at Mount Allison University by MGK, BDR, KMMS, AITH, and ARS. DA performed SIDMS analyses. CJMF provided strategic guidance. All authors contributed to the article and approved the submitted version.
