# Supplementary material for: Single hair analysis by X-ray fluorescence spectrometry detects small changes in dietary zinc intake: A nested randomized controlled trial
Source: Front Nutr. 2023 Mar 24;10:1139017. doi: 10.3389/fnut.2023.1139017 (PMC10080032; doi:10.3389/fnut.2023.1139017)

**Supplementary Figures**

Supplementary Figure S1. Correlation between the integrative and peak counts methods for measuring the sulphur content of a single hair strand using XRF

● 5-minute integrative method vs peak sulphur counts, y=18.684x - 426.47, R^2^ = 0.853

● 3-minute integrative method, vs peak sulphur counts, y= 7.3707x + 40.964, R^2^ = 0.264

Supplementary Figure S2. Correlation between the integrative and peak counts methods for measuring the zinc content of a single hair strand using XRF


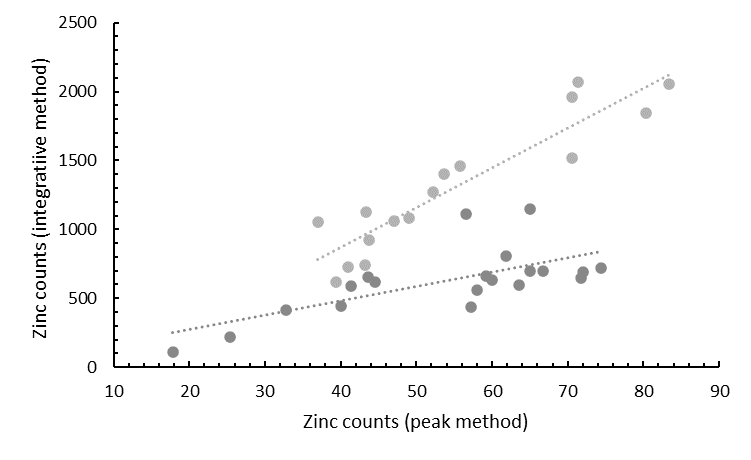


● 5-minute integrative method vs peak zinc counts, y= 28.985x – 290.55, R^2^ = 0.853

● 3-minute integrative method, vs peak zinc counts, y= 10.392x + 63.613, R^2^ = 0.468

Supplementary Figure S3

An energy spectrum from a typical 3 minute (a) and 5 minute(b) measurement trial obtained at MTA, showing Counts against Energy (keV). The detection peaks of the K X-rays from sulphur, Kα X-rays from zinc, and Kß X-rays from zinc are labeled. Given the detector resolution, it is not possible to distinguish the Kα and Kß peaks as separate peaks for sulphur

(a)


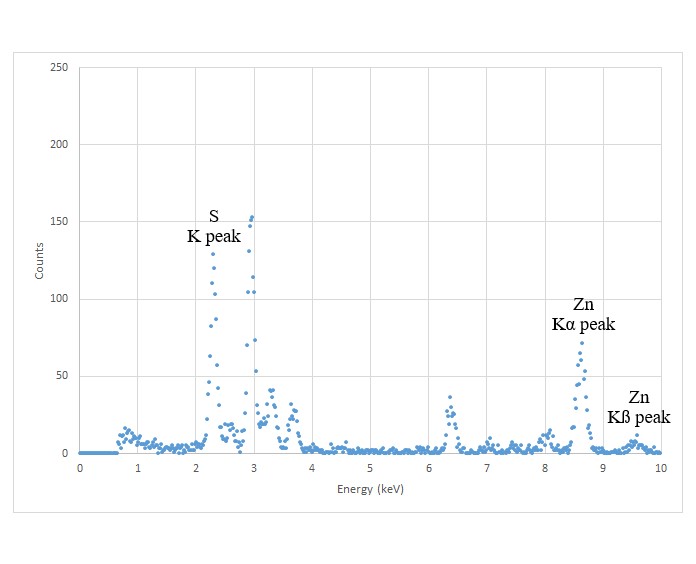


(b)


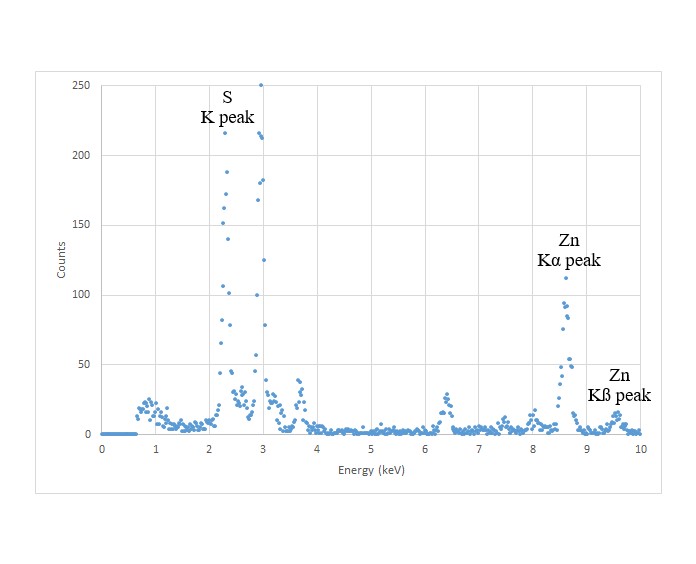


Supplementary Figure S4. Correlation between Zn:S vs [Zn] by SIDMS (µg/g), using the integrative method for Zn and S counts by XRF, y= 0.0013x + 0.4396, R^2^=0.304


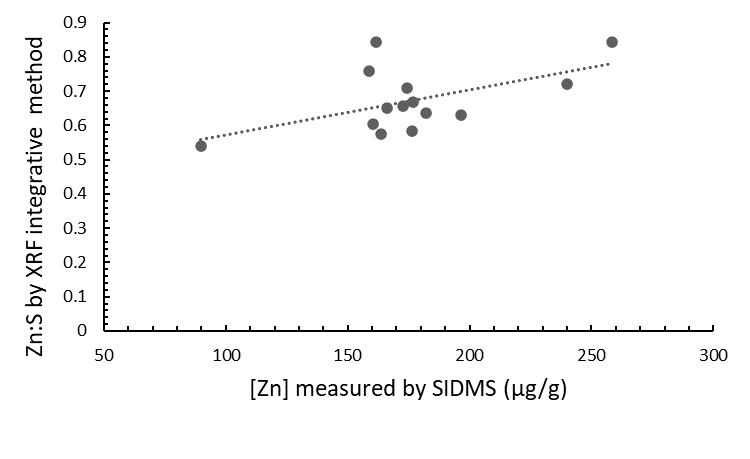


Supplementary Figure S5. Correlation between Zn:S vs [Zn] by SIDMS (µg/g), using the peak counts method for Zn and S counts by XRF, y= 0.001x + 0.2611, R^2^ = 0.407


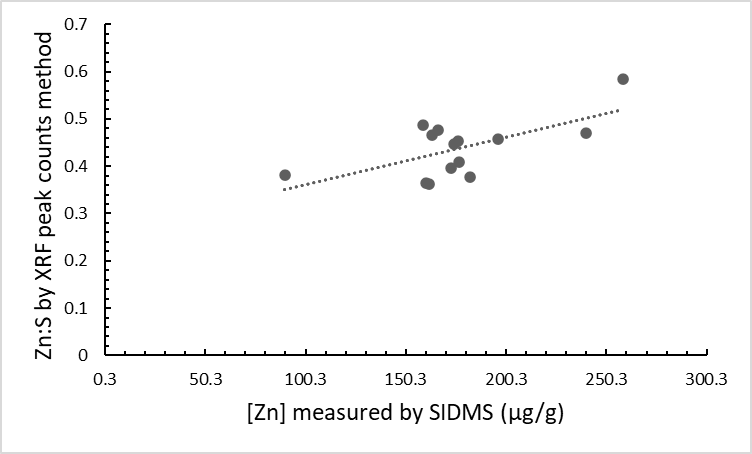

Supplement: Supplementary file 1 [file Data_Sheet_1.docx]
